# The organisation of the stress response, and its relevance to chiropractors: a commentary

**DOI:** 10.1186/1746-1340-14-25

**Published:** 2006-10-18

**Authors:** Katie Hardy, Henry Pollard

**Affiliations:** 1 ONE Research Foundation, Encinitas California, USA; 2 Macquarie Injury Management Group, c/o PO Box 448, Cronulla NSW, 2230, Australia

## Abstract

The stress response is a natural reaction by the body, against potentially harmful stimuli to enhance the chance for survival. Persistent activation of the stress response can cause changes to homeostatic mechanisms. The study of stress neurophysiology, in the evaluation of the manifestation of disease in the body, suggests that these chronic changes have detrimental effects on sub cortical structures. Furthermore, there is much scientific support for the notion that chronic activation of supraspinal systems will lead to maladaptation of homeostatic mechanisms, causing the impairment of processes within the body, and ultimately leading to visceral disorders. The chiropractic profession for many years has alluded to chronic change of neurophysiological pathways as a potential explanation of visceral disorders, but the profession has typically described these in terms of somatovisceral or viscerosomatic reflex activity. Change in supraspinal neurophysiological efferent activity is increasingly being used to explain "stress" related disease. The chiropractic profession should consider investigating such stress responses by conducting spinal manipulative therapy trials that evaluate supraspinal effects of manipulation. Such research may help elucidate key mechanisms associated with the change of visceral disorders noted by some chiropractors following manipulative therapy.

## Background

Walter Canon offered the first model of homeostasis as the "coordinated physiological processes which maintain most of the steady states in the organism" [1-3], and further focused on the "sympathetic nervous system as an essential homeostatic system that served to restore stress-induced disturbed homeostasis and to promote survival of the organism". From work conducted during the 1930's to 1950's, Hans Selye introduced the concept of stress as a medical and scientific entity, depicting a pathological triad elicited by numerous stressors. Sleye then employed this defined theory of stress as "the non-specific response of the body to any demand" [4]. Selye proposed that the human body had a finite amount of adaptable energy, and opined that a stressor whether pleasant or not, was irrelevant because any type of stress required adaptation to manifest. The important criterion was the intensity of the demand, and whether the body could meet that demand with an appropriate response. This cognitive response came to be known as the "fight-or-flight" response [5] and involved the activation of necessary physiological and behavioral responses for survival [6]. These responses are often referred to as 'stress responses' and include the activation of the hypothalamic-pituitary-adrenal axis and sympatho-adrenal system, resulting in the consequential secretion of multiple hormones including corticotrophin releasing hormone, adrenocorticotropin hormone, cortisol, norepinephrine and epinephrine [7]. Once the stress response is activated, behavioral and physiological changes lead the way for the organism to adjust homeostasis within the body, and increase its chances for survival [8].

It is in times of sustained or repeated activation that the stress response may alter [7]. Due to the intricate nature of the above systems, systematic changes can cause dramatic effects on organs, which otherwise would be activated in advantage for the organism [9]. Repeated stimulation of hormones, and neurotransmitters may render target tissues resistant, instigating the cascade into disease and illness [10]. Many pathological processes, such as chronic pain disorders, immune disorders, cardiovascular disorders, metabolic disease and behaviour disorders, may be the result of chronic activation of the hypothalamo-pituitary-adrenal axis and sympatho-adrenal system, affecting tissues or biological pathways, contributing to the global nature of disease [11,12].

As stress becomes more prominent in society, trying to understand mechanisms with which it manifests in the body, and potential treatment for these manifestations is imperative to the development of effective chiropractic treatment strategies. This commentary attempts to address the development of disease within in the body due to chronic stress activation. It discusses the anatomy, physiology and the relationship of adverse chronic stress activation on systems within the body. This is followed by discussion of how these variables integrate and are potentially affected by the application of manipulative therapy.

## Discussion

### Neuroanatomy

**Figure 1:**
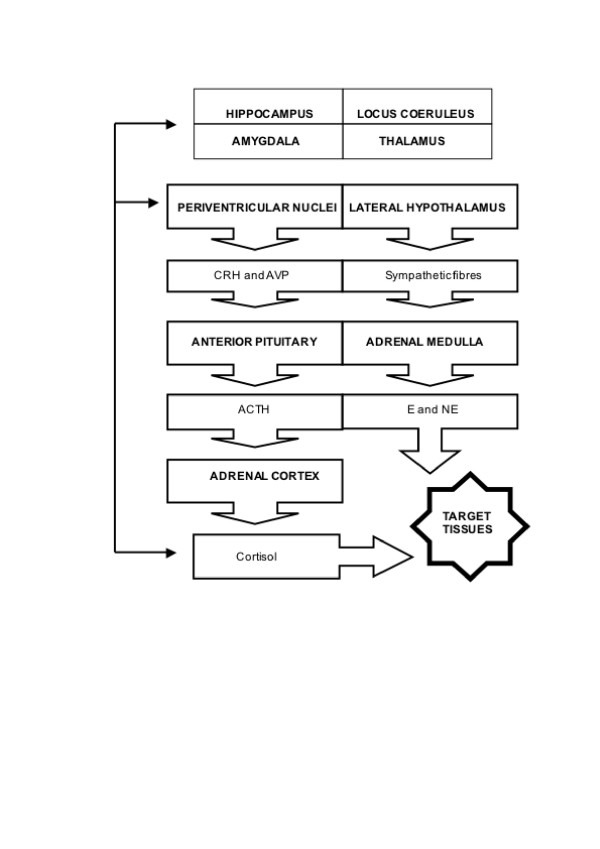
A schematic representation of the stress response. *CRH*, corticotrophin releasing hormone; *AVP*, arginine vasopressin; *ACTH*: adrenocorticotrophin releasing hormone, *E*: epinephrine, *NE*: norepinephrine.

This section will outline the neuroanatomy of the stress response, focusing on input and output pathways. As there are multiple brain structures concerned in the organization of the stress response, these systems are intricately related. A schematic representation of the stress system is presented in Figure 1.

#### Hypothalamic afferents

Nearly all stress-related information projected to the hypothalamus is congregated to the lateral hypothalamus, where combinations of numerous ascending and descending fibres are integrated from areas including the limbic system, medial hypothalamus, and the autonomic nervous system containing with thousands of interneurons [13].

Numerous *viscerosensory *signals arise from glossopharyngeal (IX) and vagal (X) cranial nerves in the spinal cord and from the lower brain stem, which project to the nucleus of the solitary tract (NTS), and terminate via multisynaptic pathways at the hypothalamus [14]. The NTS is the first region in the central nervous system that processes information about visceral, cardiovascular, respiratory functions as well as taste. Neurons of the NTS project to the paraventricular nucleus, and other hypothalamic nuclei among other destinations [15]. The NTS has other viscerosensory fibres terminating on catecholaminergic neurons, which then project to the hypothalamus [14]. *Somatosensory *signals reach the hypothalamus directly via the spinohypothalamic tract [16], by axon collaterals of fibres of the spinoreticulothalamic and/or spinothalamic tracts in the spinal dorsal horn [17], or through the activation of the brainstem catecholaminergic system [14].

Ascending medullary viscero- and somato-sensory neurons have been associated as carriers of autonomic signals to the hypothalamus. Signals may directly project to the hypothalamus, or indirectly through secondary autonomic centres, such as the parabrachial nuclei [13].

#### Hypothalamic efferents

The output system involves the recognition of the stress response, and engages both the neuroendocrine and neuronal pathways [13]. The higher centres consisting of the cerebral cortex, limbic system and hypothalamus are neuronally connected with the brain stem, autonomic and sensory centres, as well as interconnected with each other. The higher centres do not have connections to the periphery, though they are able to indirectly and bilaterally influence sympathetic and parasympathetic preganglionic neurons via the efferent paraventricular pathway [18].

The hypothalamus is able to exert effects via neurohumoral pathways through the pituitary, autonomic effects via neuronal pathways to preganglionic neurons, and able to exert both parasympathetic and sympathetic effects through the medulla oblongata and spinal cord [14].

The four predominant nuclei containing descending hypothalamic-autonomic fibres include the paraventricular, arcuate, perifornical and dorso-lateral hypothalamic nuclei. The hypothalamic paraventricular nucleus (PVN) is a central site in the complex of interacting systems controlling the stress response [19]. It is the foremost source of descending hypothalamic pathways to autonomic centres, with fibres arising from dorsal, posterior and lateral parvicellular subgroups containing a variety of putative neurotransmitters. Toth et al. [20] investigated the decussations of descending fibres of the PVN by using *vulgaris*-leucoagglutinin in intact brain stem operated rats. Paraventricular fibres descend by the length of the brainstem and spinal cord via two descending tracts: one from the lateral hypothalamus, along the lateral lemniscus and from the pons, moving ventrolaterally and running into the ventrolateral medulla. Some of these fibres loop dorsomedially in the caudal ventrolateral medulla to innervate the NTS, and dorsal motor nucleus of the vagus nerve (X). Secondary fibres run periventicularly and join the above tract at the pontine level [21]. Toth et al. [20] found descending fibres of the PVN decussate supramamillary, at the pontine tegmentum, at the commissural part of the NTS (major cross over area), and at Lamina X of the thoracic spinal cord levels. Termination of these pathways has been confirmed in the NTS [22], A1/C1 catecholaminergic cell groups [18], pre-sympathetic (rostroventrolateral) medulla and sympathetic (IML) neurons [23]. The PVN has been implicated in a variety of behaviors including feeding, thirst and cardiovascular mechanisms as well as in the organization of autonomic and endocrine responses. The arcuate nucleus projects to preoptic LHRH neurons, and is involved in the regulation of gonadotrophin secretion and sexual behaviour during the female reproductive cycle. The dorso-lateral hypothalamic nuclei project fibres to lateral parts of the brain stem, with numerous fibres terminating in the caudal brainstem lateral tegmentum, including the locus coeruleus, parabrachial nuclei, nucleus subcoeruleus, and the solitary and dorsal vagal nuclei [24].

With arrangements of ascending and descending fibres from various areas including the limbic system, medial hypothalamus, and the autonomic nervous system, the hypothalamic system is able to exert effects on the neuro-endocrine and neuronal pathways, as well as indirect influences to the periphery, playing an extremely important role in mediation of the stress response.

#### Limbic afferents

The limbic system is located in the boundary between the telecephalon and the diencephalon. Environmental stimuli, posing as external stressors, are recognized by specific sensory receptors systems, which transmit information via the nucleus of the solitary tract to respective sensory areas of the thalamus from spinal or cranial sensory neurons. These inputs include mechanoreceptors. [25]

Sensory information is transmitted to the amygdala through direct thalamo-amygdala connections, or indirectly though thalamo-cortico-amygdala connections [27]. The lateral nucleus of the amygdala receives and processes information from both pathways, and then projects to central, basal and basal accessory nuclei of the amygdala [28], as well as to the hippocampus, orbitofrontal cortex, cingulate [29] and via the reticular activating system to the sensory cortex [30,31]. The sensory cortex then directs information directly to the amygdala, or via the hippocampus and then to the amygdala. [32].

The hippocampus does not receive information involving individual sensory stimuli, but more general contextual cues [27]. Contextual cues can be seen as tasks performed in a predefined movement. When a cue is available, the motor system forms an internal model that uses both serial order and target direction to program motor commands [33]. The hippocampus thereby receives information on a universal basis, participating in declarative memory function, and integrating important contextual elements such as the time and place of events. This is important for retrieval and utilization of stored information into the future [29]. External stimuli present information to sensory processing systems of the neocortex, which creates a memory context through primary and higher order sensory cortices [27]. These systems project to association cortices, such as the parieto-temporo-occipital and prefrontal, and then to the transitional cortex, including entorhinal, parahippocampal and perirhinal areas of the limbic system, where different memory contexts are incorporated. The entorhinal cortex projects these incorporated contexts to the hippocampus where even more complex contexts are established [34]. Back through the same pathways, the hippocampus projects to the neocortex and forward to the amygdala and paraventricular nucleus (PVN) of the hypothalamus [30]. Retrieval and utilization of stored information is carried out through the hippocampus and other related cortical areas, and projected to the amygdala, which may trigger a stress response of context memories, even without external events [35].

#### Limbic efferents

Individual stimuli presenting as external stressors, or emotional evaluation of internal stressors are fundamentally analyzed by the amygdala [34]. Therefore the central nucleus of the lateral amygdala is involved in the coordination of stress behaviour and modulating memory consolidation [6], as well as controlling neuroendocrine and autonomic responses [36,37]. The regulation of these systems occurs through numerous connections including direct projections through the bed-nucleus of the stria terminalis to the hypothalamus [8], which projects to the lateral hypothalamus to mediate the activation of sympathetic component of the ANS [36]; projections to the dorsal motor nucleus of the vagus are involved in the activation of the parasympathetic component of the ANS [37]; projections to the NLC and ventral tegmental area are involved, respectively in the activation of the noradrenergic and dopaminergic systems [38] and projections to the midbrain central gray mediate certain behavioural responses and importantly direct projections to the PVN of the hypothalamus [39].

The hippocampus also regulates the neuroendocrine stress response by inhibition of the HPA axis through glucocorticoid negative feedback [40]. Network functions within the hippocampus are altered by persistent corticosteroid actions, resulting in decreased accuracy and reliability of contextual memories [6]. In a situation where a decision needs to be made whether or not there is a stress, this decreased accuracy and reliability prevents access to important information. The amygdala has neuronal projections to the paraventricular nucleus of the hypothalamus, and this amygdalo-hypothalamic pathway is believed to influence the activity of the neuro-endocrine hypothalamic-pituitary system, and behavioural functions under a range of physiological conditions [41]. This amygdalo-hypothalamic pathway is believed to perform an essential role in the adreno-cortical response to a number of somatosensory stimuli [42].

Thus, the limbic system is actively involved in the body's stress response and this stress response is affected by memory and emotion. It is important these interactions are considered in terms of the known associations of chronic pain and psychosocial variables [43-45]. It is likely that such associations should receive greater attention in diagnosis and treatment by chiropractors, particularly those managing chronic pain syndromes. New techniques such as the neuroemotional technique (NET) that claim to consider such variables in the diagnosis and management of pain should endeavour to measure variables of the stress response to support rhetoric that their management approaches can manage chronic pain and disease by the application of techniques associated with cognitive and behavioural principles.

### Neurobiology

Mechanisms used by the body to respond to stressful stimuli are illustrated below. The intricate biology of the stress response is depicted via numerous pathways working together to function at a systemic level. These systems are activated under different mechanisms, controlling areas of the body as means of survival. The principal physiological responses to stress are mediated by the sympathoadrenal system, and the hypothalamo-pituitary-adrenal axis [46].

#### Sympatho-Adrenal System (SAS)

The autonomic nervous system is a rapidly responding mechanism, controlling numerous systems including cardiovascular, gastrointestinal, respiratory, renal and endocrine which are under control of the sympathetic, parasympathetic or both nervous systems [47]. Activation of the SAS system functions to reduce blood flow, reduce activity of the gastro-intestinal system and reproductive organs, as well as mobilise energy to the brain, heart and muscles [48]. It does this via synapses of sympathetic preganglionic fibres in the intermediolateral column of the spinal cord with the postganglionic sympathetic neurons innervating the smooth muscle [49]. This evolutionary mechanism has evolved to create quick compensatory changes in homeostasis for intense physical activity, by increasing the capacity of the 'fight or flight' reaction and therefore promote survival.

During an antecedent event, activation of the SAS system (locus coerulus/norepinephrine/sympathetic nervous system) evokes the release of noradrenaline and neuropeptide-Y from postganglionic nerve terminals, while preganglionic innervation of the adrenal medulla results in an increased secretion of adrenaline and dihydroxyphenylaline (DOPA) [50]. Activation also results in the increased secretion of Interleukin-6, an important cytokine that connects the stress system and various immunological and inflammatory processes [6].

#### Hypothalamic-Pituitary-Adrenal Axis (HPA Axis)

The hypothalamo-pituitary-adrenocortical (HPA) axis plays a fundamental role in adaptation of the organism to homeostatic challenge, and should be thought of as the body's energy regulator, as it is ultimately responsible for controlling virtually all of the hormones, nervous system activity, and mineral homeostasis [13]. Activation of the HPA system results in secretion of glucocorticoids, which act at numerous levels to redirect bodily energy resources [51]. These hormones are recognized by glucocorticoid receptor molecules in numerous organ systems, and act by genomic mechanisms to modify transcription of key regulatory proteins [51].

The HPA axis is readily activated by stressful stimuli [46]. Sensory information reaches the cortex via the thalamus and is conveyed to the central amygdaloid nucleus of the amygdala [8]. It responds by providing the stimulus to cortico-releasing hormone (CRH) neurons in the paraventricular nucleus of the hypothalamus to increase the secretion of principal neuropeptide CRH and arginine vasopressin (AVP) into the hypophyseal portal bloodstream. These secretions are thence transported to the anterior pituitary gland [52]. The corticotrophin producing cells of the anterior pituitary synergise CRH and the AVP through CRH-RI and VIb receptors respectively to increase expression of the adreno-corticotropin hormone (ACTH) precursor, and further promote the release of ACTH into the systemic circulation [53]. Upon release, ACTH stimulates the zona fasciculata cells of the adrenal cortex to release synthesized glucocorticoid (cortisol in humans) and mineralocorticoid hormones (principally aldosterone) [54,55]. Via a delicate negative feedback loop, glucocorticoids control the termination of the stress response via inhibitory control of the production and release of CRH and ACTH at the level of the hypothalamus and pituitary respectively, via transmitter systems projecting to the hypothalamus [56]. Glucocorticoids activate steroid receptor mediated transcriptional regulation and membrane receptors, inducing changes which serve to mobilize energy and inhibit other potentially costly reactions to stress. Glucocorticoids promote gluconeogenesis, increase blood pressure and suppress aspects of immune and reproductive function [57]. In addition Inhibition of the ACTH response occurs through the glucocorticoids binding to receptors in the hippocampus, which control CRH production and limiting the period of exposure to the stress response, therefore minimizing the catabolic immunosuppressive and anti-reproductive effects of glucocorticoids [6].

Catecholamines are involved in these pathways as chemical messengers. Brainstem catecholaminergic and non-catecholaminergic neurons, through collateral branching inputs may provide coordinated signaling of visceral input to rostral forebrain sites. This may lead to a synchronized response of the CNA and PVN for the maintenance of homeostasis [58]. Thus, emotion and memory effects mediated via these centres have the potential to cause dysfunctioin in the viscera. Such dysfunction should be considered in the management of chronic pain and disease states

#### Magno- and Parvo- cellular system

The magnoceullular neurons of the supraoptic (SON) and PVN, along with scattered clusters of cells between the two nuclei, comprise the hypothalamo-hypophyseal system. These cells send oxytocin and vasopressin containing fibres to the posterior pituitary where these substances are released into the peripheral circulation. Vasopressin is an antidiuretic hormone (ADH) and is released in response to changes in osmotic pressure of circulating blood or extra-cellular space. ADH controls water balance, in particular retention of water regulated in the distal tubules of the kidneys.

### Implications of the stress response on the body

Everyday interactions with the environment inevitably expose the body to a wide range of stressful stimuli. The stress response has evolved for efficient functioning of the neuro-endocrine and neuronal pathways to play a vital role in adaptation of the body to homeostatic challenges brought to bear on it. It is during times of repeated, chronic activation that dysregulation of the stress response may lead to manifestation of disease of the body. Chronic stress may lead to physiological systems within the brain and body fluctuating to meet internal or external demands, causing deterioration, and leading to maladaption [10]. Physiological systems such as the central nervous system, reproductive system, cardiovascular system, metabolic system and immune system are all involved in survival and adaptation. When stress cannot be normalised (enabled, disabled or decreased), it may become detrimental to health. The detrimental effects of stress may manifest in several system wide disorders such as: behavior/mood disorders of substance abuse and depression [59,60]; cardiovascular disorders such as hypertension, atherosclerosis and cardiovascular disease [61]; metabolic diseases such as insulin resistance/metabolic X syndrome and obesity ([52]; immune disorders that include chronic inflammatory processes, autoimmune diseases [62]; or vulnerability to these or other diseases [63]. Thus, inefficient activation of the stress system may have destructive effects bodily functions and these effects may contribute to the onset and maintenance of organ/tissue dysfunction.

#### Glucocorticoids

Glucocorticoids, adrenal hormones secreted during stress, are seen as key hormones, which are able to permit, stimulate or suppress the stress response [52]. The primary glucocorticoid in humans, cortisol, is secreted continuously by the adrenal cortex in a diurnal pattern, with early morning peaks and evening troughs, but secretion can increase dramatically in the dynamic setting of environmental stressors. Its secretion can adversely affect many bodily functions [7]. Glucocorticoids restrict circulating CRH and ACTH levels, preventing inappropriate steroid exposure following a brief episode of stress. Network functions within the hippocampus are disturbed by persistent glucocorticoid activation, triggering secretion outside the normal physiological range, resulting in atrophy of the human hippocampus [64], and alteration of network functioning [65]. Damage associated with changes in the regulation of the HPA axis cause deficits in memory, cognition and learning [30], and decreased accuracy and reliability of contextual memories [6], thus contributing to behavioural adaptations to the response to stress. Prolonged elevations of glucocoticoid levels cause diseases such as Cushings Syndrome [67], or contribute to precipitation of disease such as major depressive disease [10], Alzhimers Disease [66], post-traumatic stress disorder [68] and recurrent depressive illness [62].

Uno et al. [69] provided the first evidence that chronic stress resulted in glucocorticoid hypersecretion, resulting in neurodegeneration of the primate after a retrospective, neuropathological study was performed on eight vervet monkeys. At death the monkeys were found to have multiple gastric ulcers. Compared with controls euthanized for other research purposes, ulcerated monkeys had marked hippocampal degeneration that was apparent both quantitatively and qualitatively, and was detected both ultrastructurally and at light-microscopic level. It was discovered that in ulcerated monkeys which appeared to have been subject to sustained social stress, perhaps in the form of social subordinance in captive breeding groups, and which also had had significantly high incidences of bite wounds at death, these monkeys had hyperplastic adrenal cortices, indicative of sustained glucocorticoid release [69].

Excess glucocorticoids, as the end effect of chronic stimulation of the HPA axis, may constitute a base for pathophysiological consequences in the periphery of the body. This includes potentiation of sympathetic nervous system-mediated vasoconstriction, proteolysis and lipolysis [70], energy mobilization (glycogenolysis) in the liver [41], and processes such as reproductive, metabolic and immune functions. Many such changes have often been attributed to spinal based reflex changes associated with chronic somatic dysfunction.

#### Reproductive function

Systems activated by stress can influence reproduction at the hypothalamus, pituitary gland or gonads. There are close relations with the sustained activity of the end-product of the HPA axis and suppressed steroid sex and growth hormones [57]. The first wave of hormonal mediators of the stress response stimulates the production of hypothalamic CRH, which inhibits gonado-tropin releasing hormone (GnRH). Via the release of somatostatin, hypothalamic CRH also inhibits growth hormone (GH), thyrotropin-releasing hormone (TRH) and thyrotropin secreting hormone (TSH) secretion thereby suppressing growth, reproduction, and thyroid functions [71]. Glucocorticoids directly inhibit the pituitary gonadotropin, GH and TSH secretion, causing target tissues of sex steroids and growth factors to become resistant [7]. Increased glucocorticoid secretion significantly reduces peak luteinizing hormone (LH) inhibiting the effect of glucocorticoids on the pituitary gonadotroph [72]. Furthermore, suppression of the growth, reproduction and thyroid functions arises from glucocorticoid ability to suppress the 5' deiodinase, which functions to convert virtually inactive tetraiodothyronine (T4) to triiodothyronine (T3) thereby causing s stress induced state of hypothyroidism [73].

The female reproductive system is regulated by the hypothalamic-pituitary-ovarian axis. The principal regulator of the hypothalamic-pituitary-ovarian axis is GnRH [74]. GnRH stimulates pituitary follicle stimulating and LH secretion and, subsequently, estradiol and progesterone secretion by the ovary. When activated by stress, the HPA axis exerts an inhibitory effect on the female reproductive system. CRH and CRH-induced proopiomelanocortin peptides such as β-endorphin inhibit hypothalamic GnRH secretion [75]. In addition, glucocorticoids suppress gonadal axis function at the hypothalamic, pituitary and uterine level [44,76]. Furthermore, glucocorticoids inhibit estradiol-stimulated uterine growth [76]. These effects of the HPA axis are responsible for the "hypothalamic" amenorrhea of stress, which is observed in anxiety and depression, malnutrition, eating disorders and chronic excessive exercise, and the hypogonadism of the Cushing syndrome [77]. On the other hand, estrogen directly stimulates the CRH gene promoter and the central noradrenergic system [50], which may explain women's mood cycles and manifestations of autoimmune/allergic and inflammatory diseases that follow estradiol fluctuations.

#### Metabolic function

Stress is understood to contribute to the pathogenesis of disease, and under chronic conditions contributes to disease by impairing the body's ability to correctly control normal responses of the body. Increased glucocorticoid secretion causes resistance of growth hormone from osteoblastic cells of bones, inhibiting osteoblastic activity that renders them osteoporotic [73]. Chronic activation of the stress response can lead to the promotion of visceral adiposity, and is achievable by glucocorticoids stimulating hepatic gluconeogenesis, and inhibiting insulin activity on skeletal muscles and adipose tissue respectively [7]. In the abdominal region, fat cells have a higher density of corticosteroid receptors, with cortisol increasing receptor sensitivity metabolism of target fat cells, thereby increasing the storage of fat in this area [79]. Furthermore, chronic stress suppresses growth hormone, leutinising hormone, testosterone, TSH and T3 instigating insulin resistance/hyperinsulinemia and dyslipidemia [80]. This leads to the exertion of a complex and long-lasting effect involving increased SNS activity and increased cortisol and epinephrine secretion which influences non-genetic factors on the origins of type I diabetes [81] and hampers the control of Type I and Type II diabetes [82]. Here we see a demonstration of the manifestation of the "Metabolic Syndrome". As discussed by Chrousos [7] and Girod and Brotman [83], situations associated with chronic activation of the HPA axis may derive some of their associated cardiovascular risk from untoward glucocorticoid effects, leading to myocardial infarction and atherosclerosis. Raadsheer et al., [84] investigated higher circulating glucocorticoid levels, and impaired suppression of cortisol in depressed patients and found that many exhibited symptoms of increased glucocorticoid tone as discussed above, such as central obesity, menstrual irregularity, immunosuppression and osteoporosis. Chrousos [6] stated that stress-induced hypercorticolism, visceral obesity, and other related sequelae have the potential to increase the all-cause mortality of affected subjects by 2–3 times, and shorten life expectancy by several years.

#### Immune disorders

Glucocorticoids have been clinically used to control autoimmune disease, and inflammation, as well as organ donation rejection for many decades [85]. The role of glucocorticoids is to inhibit the production of proinflammatory cytokines such as tumor necrosis factor (TNF)-**A**, interferon (IFN)-Y and interleukin-12 (IL-12), and to stimulate the production of anti-inflammatory cytokines such as IL-10, IL-4 and transforming growth factor (TGF)-B [86]. During chronic inflammatory stress, interleukin-6 (IL-6) plays a major role of the endocrine cytokines in the immune stimulation of the HPA axis [73]. Chronic activation of the stress response has been found to impair immune functions, and delay the healing process [79]. The role of the HPA axis is crucial to regulating the severity of autoimmune disease, though the cause remains obscure. In the absence of corticosteroids the immune system is unrestricted and its activation by either acute or chronic immune challenge is likely to be fatal [87].

#### Neuromuscular disorders

Predictable disorders such as low back pain, tension headache and even rheumatoid arthritis may be a result of repeated activation of postural musculature during chronic flight-or-flight responses [88]. Jacobson [89] first argued that proprioceptive impulses could be found in conditions of high musculoskeletal tension. It was hypothesized that if such tension was combined with high levels of sympathetic activity, it could contribute to anxiety reactions. Krantz et al. [90] investigated different physiological responses to stress, as well as surface electromyography of the trapezius muscle. They found significant association between sympathetic arousal and electromyography activity, which is of importance when understanding the relation between musculoskeletal disorders and stressful situations. This is of particular interest in the treatment of work-related pain disorders in psychosocial stress syndromes as they may cause the development of pain disorders [91,92]. Therefore, chiropractors may require additional interventions to manage all aspects of chronic pain syndromes presenting to them. Combined psychological and manual interventions may provide the most appropriate healing of chronic conditions of the musculoskeletal neurohormonal systems commonly associated with chronic stress and disease. Further research is needed on the physiological and psychological effects of stress and its manifestation on the musculoskeletal system, as well as the effects of manipulative treatment on physiological and psychological functions. Emphasis should be given to the potential measurement of the stress response on various bodily systems after the application of manipulative therapy in normal and disease states.

### Relevance to chiropractic

Chiropractors treat conditions of a neuromusculoskeletal and non-neuromusculoskeletal nature [93]. The majority of conditions treated by chiropractors are neuromusculoskeletal [94]. Much controversy exists about the role of chiropractic in the management of the non-neuromusculoskeletal conditions [95]. These non-neuromusculoskeletal conditions are sometimes referred to as "type O" conditions and the neuromusculoskeletal conditions are sometimes referred to as "type M" conditions. To justify the relevance of the management of the "type O" condition, the chiropractic profession has usually cited the presence of the somatovisceral and viscerosomatic reflexes as being integral to both the cause and potentially the management of the "type O" disorder [96,97]. Despite this dependence on the existence of these reflexes to justify a philosophical approach to management, little evidence exists to warrant the continued support of this justification.

The literature supports the existence of somatovisceral and viscerosomatic reflexes [98-100], but there is little or no evidence to support the notion that the spinal derangements (often referred to as subluxations by chiropractors) can cause prolonged aberrant discharge of these reflexes. Equally unsupported in the literature is the notion that the prolonged activation of these reflexes will manifest into pathological state of tissues, and most relevantly, that the application of spinal manipulative therapy can alter the prolonged reflex discharge or be associated with a reversal of the pathological degeneration of the affected reflexes or tissues [101,102]. The evidence that has been amassed is largely anecdotal or case report based [102-104] and it has attracted much intra disciplinary debate [102,105,106] because of its frequent association with certain approaches to management (largely described as being traditional or "philosophical" in nature).

Traumatic stress of a physical nature is known to manifest in changes to limbic, memory and other relevant stress centres in the brain including the hypothalamus and pituitary [107]. Whilst still controversial in management and diagnosis [108] conditions such as post traumatic stress disorder may be the linking mechanism for the manifestation of psychosocial variables often noted by chronic pain researchers [109-113]. If this supposition is true, would a purely mechanistic treatment approach be appropriate for its cost effective management?

Despite prolonged debate, very little consideration has been given to other potential mechanisms (and solutions) for the presence (and resolution) of the "type O" condition. A recent review has detailed that the somatovisceral reflex is a short term effect (millisecond to seconds in duration) when compared to supraspinal influences on the spinal cord which can be weeks to months in duration [102]. The review reasoned that the chiropractic profession should consider supraspinal factors in the generation and management of chronic pain states [102]. This conclusion is particularly pertinent on the now known association of psychosocial variables in chronic pain and disease [102], and the fact that many of the conditions treated by chiropractors whether type O or M are of a chronic nature [95]. However, the call to look at non-spinal non mechanical aspects of management has not been well received by the profession to date as evidenced by the continued predominantly mechanistic approach to management.

Chronic pain is associated with stress [114]. It is a matter of fact that stress can affect multiple systems within the body [115], including the neuromusculoskeletal system ("type M" disorders) [101] and the non-neuromusculoskletal systems ("type O" disorders) [101].

As with all homeostatic function, individual functions often have an optimum range of function outside of which function is decreased or becomes pathological. Thus, it is plausible that too little or too much of a particular function may be detrimental to the optimal function of the organism. Chronic stress is associated with abnormal organ function and the presence of disease [116,117]. Removal of stress has been shown to rehabilitate stress induced disease [118]. Can the chiropractic treatment (spinal manipulative therapy or adjustment) reduce stress levels as a potential mechanism for improvement of the "type M" or "type O" disorders? Are the newer techniques such as neuroemotional technique [119] that incorporate elements of cognitive/behavioural principles, Pavlovian conditioning and repetition compulsion with spinal manipulative therapy effective in altering stress associated with stress related disorder? Is any potential reduction associated with peripheral or supraspinal mechanisms of action? Can the profession adapt to its primarily mechanistic paradigm to truly incorporate the biopsychosocial model of disease first proposed by Engel in the 1970's [120].

#### Stress as a mechanism of system wide disease

It seems reasonable to examine physiological systems involved in the processing of symptoms such as pain in the many conditions reported to be managed by chiropractic intervention strategies. Whilst the somatoviseral reflex has been used as an example of a possible mechanism for the cause and management of these conditions, the scope of conditions that cannot be addressed by this mechanism is still large. We contend that a higher centre system impacting on the spinal cord could better explain the diversity of conditions.

In doing so, a consideration should be given to the role of psychosocial variables resulting from the stress of various events including trauma [121]. Findings from animal studies have interestingly suggested that hormones of the HPA axis, pain-processing pathways, and autonomic nervous system may be underlying peripheral as well as central substrates of chronic pain and broad system dysfunction [122,123]. Traumatic physical or psychological stress can have enduring impact on functioning of these systems, and chiropractors manage conditions incorporating elements of these systems [124]. An impaired HPA axis could serve as a physiological mechanism of medically unexplained symptoms as well as function as a mediator between psychological distress and observed symptoms [125].

#### Chiropractic management of non-musculoskeletal conditions

Chiropractic management strategies have been used to manage or co-manage a number of non-musculoskleletal, non-malignant conditions. The pubmed database was searched in June of 2006 with the terms "chiropractic" and "case" and returned more than 589 hits. Of these, more than 40 related to non-musculoskeletal care. Some examples included: Ehlers-Danlos syndrome [126], gastroesophageal reflux [127], cervical spinal cord compression[128], congestive heart failure [129], asthma [130], cervical dystonia [131], ulnar tunnel syndrome [132] and myaesthenia gravis [133]. Unfortunately more examples of complications were returned in the pubmed database with this search string than there were examples of non-musculoskeletal treatment. The ratio appeared to be at least 3 to one.

By contrast, 416 hits were returned from the same search string on the Index to Chiropractic Literature database (ICL). There appeared to be fewer reports of negative outcomes, and the scope of the reports appeared just as broad as that presented on the Pubmed databases. Some examples include: otitis media [134], post polio syndrome [135], urinary incontinence [136], Dejerine-Roussy syndrome [137] and infantile colic [138].

It appears that the two databases present very different perspectives based on the content of their contributing journals, one (Pubmed) largely a medical database and the other largely chiropractic (ICL). This differential may explain and or reflect the different perspectives that chiropractors hold with regard to the management of non-musculoskeletal conditions when compared with their medical counterparts.

As mentioned previously, chiropractic often postulates the somatovisceral reflex as being the vehicle for the changes noted in the above conditions [101]. However, less speculation is given to the potential role of supraspinal or cortical processes in the etiology and management of such conditions. As a large body of research currently supports the role of psychosocial variables in the generation and maintenance of disease [139-145], chiropractic should look to this research to potentially explain some of the clinical observations being made and recorded in the journals.

A review by Siegrist and Marmot discusses psychosocial variables as causative, aggravating, and perpetuating factors for the stress response, as well as stress implicating elevated psychosocial variables [146]. The stress response has been associated with the propagation of numerous disorders including: dermatological [147], cardiovascular [148,149], diabetic [150], Hepatic [151], immune [152], thyroid [153], gastrointestinal [154,155], reproductive [156-158], renal [159], metabolic [160], rheumatic [161] and musculoskeletal [162,163].

Future studies could utilize a randomized controlled trial design and measure variables such as: self reported levels of pain, disability, anxiety, depression, as well as objective blood and urine based measures of stress including: proinflammatory cytokines (TNF-alpha, IL-1, IL-6, IL-8, IL-18) [121,164,165] and anti inflammatory cytokines such as IL-4 and IL-10 [122,165,166]. These tests could be cross-referenced to the expression of collagen expressed in the inflammatory reaction frequently associated with chronic disease. Research designs such as the above would provide information on the effect of chiropractic management on subjective variables of pain as well as objective measures of stress and provide insight into the mechanism of action. It is only with similar studies can the association of stress be investigated thoroughly and its relevance to chiropractic measured accurately.

## Conclusion

Sufficient evidence exists to consider stress and its mechanism, in the generation of diseases often seen by chiropractors. To date little investigation of this potential mechanism of disease and treatment has been conducted by the chiropractic profession. In a time when peak chiropractic organizations are calling for a mind-body approach to the management of chronic musculoskeletal and non – musculoskeletal disease [165], due consideration of the body of neurobiological evidence that supports the broadening of the operating paradigm within chiropractic seems warranted. Despite the call for a broadening of approaches and the embrace of such approaches by groups within chiropractic, it appears the threat to the dominant paradigm appears too great for most to adapt. The profession should consider more closely the emerging areas of study such as psychoneuroimmunology and how the development of that literature actually supports a broadening of the dominant mechanistic paradigm to reflect recent advances in science.

## Competing interests

The author(s) declare that they have no competing interests.

## Authors' contributions

KH participated in its design, constructed the literature review, and helped to draft and edit the manuscript

HP conceived of the study, participated in its design, constructed the literature review, and helped to draft and edit the manuscript. All authors read and approved the manuscript.
